# Measuring ventilation in different typologies of rural Gambian houses: a pilot experimental study

**DOI:** 10.1186/s12936-020-03327-0

**Published:** 2020-07-31

**Authors:** Jakob B. Knudsen, Margaret Pinder, Ebrima Jatta, Musa Jawara, Mahamed A. Yousuf, Amalie T. Søndergaard, Steve W. Lindsay

**Affiliations:** 1 Schools of Architecture, Design and Conservation, The Royal Danish Academy of Fine Arts, Philip de Langes Allé 10, 1435 Copenhagen K, Denmark; 2 Medical Research Council Unit, The Gambia at the London School of Hygiene & Tropical Medicine, Fajara, The Gambia; 3 Department of Biosciences, Durham University, Durham, UK; 4 London School of Hygiene & Tropical Medicine, London, UK; 5 National Malaria Control Programme, Banjul, The Gambia

**Keywords:** Ventilation, Airflow, Housing, Malaria, The Gambia

## Abstract

**Background:**

African houses are frequently too hot and uncomfortable to use a bed net at night. Indoor thermal comfort is often evaluated by measuring temperature and humidity, ignoring ventilation. This study explored ways to measure ventilation in single-roomed rural Gambian houses during the malaria transmission season and evaluated building designs that could increase airflow at night and help keep the occupants comfortable.

**Methods:**

Two identical mud-walled houses were constructed with a metal roof, three doors and closed eaves. Experiment 1 compared five methods for measuring ventilation in a building: (1) using a blower door, (2) increasing carbon dioxide (CO_2_) levels indoors using an artificial source of CO_2_ and then measuring the rate of gas decay, (3) using a similar approach with a natural source of CO_2_, (4) measuring the rise of CO_2_ when people enter a building and (5) using hot-wire anemometers. Experiment 2 used CO_2_ data loggers to compare ventilation in a reference metal-roofed house with closed eaves and badly-fitting doors with a similar house with (1) thatched roof and open eaves, (2) eaves tubes, (3) screened doors and (4) screened doors and windows.

**Results:**

In experiment 1, CO_2_ data loggers placed indoors in two identical houses showed similar changes in airflow (p > 0.05) for all three methods recording either decreasing or increasing CO_2_. Blower doors were unable to measure airflow in houses with open eaves or screened windows and the anemometers broke down under field conditions. In experiment 2, open eaves in thatched houses, screened doors alone, and screened doors and windows increased indoor ventilation compared to the reference metal-roofed house with closed eaves and badly fitting doors (p < 0.05). Eaves tubes did not increase ventilation in comparison to the reference house.

**Conclusion:**

CO_2_ data loggers proved to be a simple and efficient method for measuring ventilation in rural houses at night. Ventilation of metal-roofed houses can be improved by adding two screened doors and windows on opposite walls. Improved ventilation will result in increased thermal comfort making it more likely that people will sleep under a bed net.

## Background

Between 2019 and 2050 the population of Africa will have almost doubled [1]. This is equivalent to combining the current population of India and Africa in 30 years. Assuming that a third of the current housing stock has to be rebuilt during the same period [2], homes for up to 2 billion people will need to be built by 2050 to accommodate this growing population. To put this in perspective, that is equivalent to building housing for 7000 people per hour for the next 30 years to keep up with the population growth.

Sub-Saharan Africa’s economies have been growing rapidly in the past 20 years [3], and the region has witnessed an increase in improved housing [2]. This is good news as better houses are associated with less malaria [4]. One of the biggest changes in house design is the replacement of thatched-roofs with metal-roofs. During this process the eaves are often closed which is good for reducing mosquito entry [5] and the metal-roofs also seems to reduce survivorship of indoor-resting mosquitoes [6] as they make the building hotter during daytime. But closing of the eaves might also reduce indoor comfort as it reduces ventilation in a house [7, 8].

Indoor climatic comfort is important since if it is too hot and there is little, if any, ventilation, it is likely to reduce the number of people sleeping under a long-lasting insecticidal net (LLIN) [9] which is one of the most effective malaria control tools available [10]. The fundamental problem of designing a good house in the hot-humid tropics is one of attempting to achieve two apparently contradictory goals: to keep mosquitoes out of the house, while at the same time, maximizing the indoor ventilation in order to keep the occupants comfortable [11].

So far, there has only been one previous study that has measured ventilation or airflow in traditional African houses. This was carried out in Uganda where carbon dioxide (CO_2_) ice was left to evaporate inside a house and, after reaching a maximum concentration, the rate of decline measured using a hand-held infrared CO_2_ analyser [12]. The rate of CO_2_ decline was greater in well– ventilated houses compared with poorly ventilated houses. Whilst this was an efficient method, two people remained indoors during the measurements, which is potentially unsafe and would have affected the readings since their CO_2_ production would vary at different indoor temperatures [13, 14]. Moreover, in many parts of sub-Saharan Africa the storage of CO_2_ ice in hot and remote areas is impracticable, as is the more sophisticated methods using radioactive tracer gases [15]. In the present study, five methods for evaluating indoor ventilation were tested. Firstly, a blower door was used which is typically used for measuring airtightness in houses in Europe and North America [16, 17]. The rectangular blower door fits tightly over an existing door gap and blows air into the room through a large fan until the pressure is constant. Ventilation, or air change rate, is typically measured as Air Exchange per hour (ACH), which can be calculated knowing the volume of the house and the airflow the fan has to produce in order to reach a certain pressure for the whole building. Secondly and thirdly, the rate of CO_2_ decline was measured in buildings by increasing CO_2_ levels using a gas cylinder or human subjects, removing the source, and measuring the rate of CO_2_ decline using data loggers. Fourthly, the natural rise in CO_2_ levels indoors was measured when human subjects were introduced to the building. Finally, a hot-wire anemometer, designed to measure air flow at low speeds was tested.

In rural Gambia, there are distinct typologies of housing with traditional houses characterized by having mud walls, thatched roofs and open eaves, whilst modern houses have mud or cement block walls, metal roofs and closed eaves. In the *RooPfs* study [18], that was designed to reduce the incidence of clinical malaria in children, traditional single room mud-walled houses were modified to have metal roofs, closed eaves, with mosquito screened windows and screened doors to increase indoor air flow, to keep the occupants cooler. It is not known, however, how these architectural features affect ACH and indoor air speed (IAS). Indoor thermal comfort is obtained when a specific combination of temperature, humidity and IAS occurs [19, 20]. Whilst it is simple to measure temperature and humidity using standard data loggers, measuring ACH and IAS remains a challenge, particularly in poor rural housing in the tropics and subtropics [21]. The primary objectives of the present study were to determine an appropriate method(s) for measuring ventilation in rural African houses and then to use these method(s) to find out whether changes in building design to increase ventilation, did actually increase ventilation.

## Methods

### Study design

Two experiments were conducted in rural Gambia using two houses of an identical size and shape. The first experiment was designed to determine what methods could be used for measuring indoor airflow in two houses with metal roofs, closed eaves and badly-fitting doors. The second experiment used these methods to compare indoor airflow in the reference house in comparison with an alternative design. The study was carried out from 1st July 2017 to 18th August 2017, during the beginning of the rainy season, when high numbers of mosquitoes are present [8].

### Study area

The study site was at the Medical Research Council Unit The Gambia’s field station at Wali Kunda (13° 34.440′ N, 14° 55.471′ W) on the south bank of the River Gambia in Lower Fulladu West, Central River Region, in The Gambia (Additional file 1: Figure S1). This is an area of open Sudanian savanna with extensive rice irrigation nearby. There is an intense rainy season from June to November, followed by a long dry season. Most clinical malaria occurs between October and December [22].

### Experimental houses

Two experimental houses, positioned on a north-west to south-east axis, were constructed 10 m apart and 10 m clear of other constructions and vegetation on all sides. Houses were the average size of a single-roomed house in rural Gambia, obtained from a survey of 400 randomly selected houses in the Upper River Region of The Gambia [8]. The external base of each house was 4.20 m × 4.20 m in area and the 2.20 m high walls were constructed from sun-baked mud-blocks (each 16 cm high × 20 cm wide × 32 cm long), with a front and back door on opposite sides, perpendicular to the line of houses, each 175 cm high and 75 cm wide. The only non-traditional building components were reinforced concrete ring-beams (20 cm high) on top of the wall, which were added to prevent the mud-blocks cracking when the heavy roofs were moved, and the metal profiles used to construct the roof frame.

The study was explained in the local language to male villagers and healthy volunteers, aged over 15 years who provided signed-witnessed consent, were recruited to the study. Two volunteers in each house slept in separate beds under an intact long-lasting insecticidal net (Olyset, Sumitomo Chemical, Japan) from 21:00 h to 06:00 h.

### House typologies

**Fig. 1:**
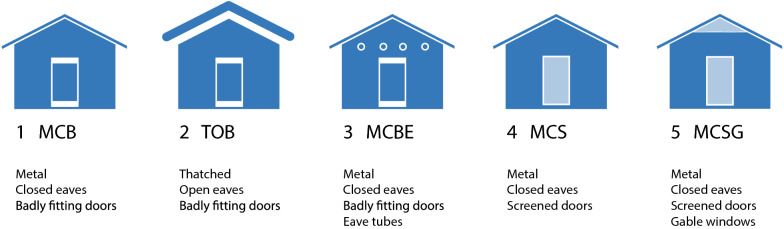
House typologies

There were five typologies of single-roomed houses used in the study (Fig. 1). Firstly, the most common typology of housing in rural Gambia is a metal-roofed house with closed eaves and two badly-fitting doors served as the reference (MCB). Secondly, the traditional Gambian house with a thatched-roof, open eaves and two badly-fitting doors (TOB). Thirdly, a metal-roofed house with closed eaves, two badly-fitting doors and four eaves tubes (MCBE). Fourthly, a metal-roofed house with closed eaves and two screened doors (MCS). Lastly, a design based on a ventilated house used in the Roo*Pf*s trial [18], which had a metal-roof, closed eaves, two screened doors and screened windows (MCSG).

Houses with open eaves had a 3 cm gap between the top of the wall and the roof and those with closed eaves were blocked with a mixture of broken mud blocks and clay mortar. Traditional doors were constructed from a single panel of corrugate galvanized steel pinned to a wooden frame (2 cm × 2 cm). To simulate poorly-fitting doors, which are common in villages, we made a 2 cm gap along the top and bottom of each door. Screened doors were made of 25 mm square steel profiles treated with anti-corrosion paint. Two screened panels made from polyester netting (2 × 2 mm mesh) (Additional file 1: Figure S3) were placed at the top and bottom of the door, each 75 cm wide and 60 cm high and fixed to the steel frame with flat bars and bolts. Both houses had a third door with an extra tightly-fitting steel door mounted with rubbers seals in order to prevent any ventilation around the door. Eave tubes were locally made from 15 cm diameter polyvinyl chloride pipes with polyester netting (2 × 2 mm mesh). Four eave tubes were placed at a height of 180 cm on each of the two facades without gables. The ventilated metal-roofed house had two screened doors and two triangular screened windows (200 cm wide and 45 cm high at the apex), constructed with wooden frames (50 mm x 50 mm) and mosquito screening, and positioned in the gable ends of the building. Thatched roofs were pyramidal in shape and 3.6 m high and metal roofs, saddle shaped and 3.1 m high. The houses with thatched roofs had an internal volume of 37 m^3^ and the metal roof houses had 38 m^3^. Floors were beaten mud.

### Experiment 1. Ventilation tests

Four methods for measuring ACH and one method for measuring IAS were tested.

#### Method A. Fan pressurization (blower door test)

ACH was estimated using a standard blower door (Model DB B, BlowerDoor, Serial #DB-CE1475, calibrated 17.10.2016, Certificate 8-DB-CE1475-10-17-16, The Energy Conservatory, Minneapolis, USA), based on a standard methodology [16, 17, 23]. The tightly-fitting steel door was opened for this experiment only and the blower door fitted in the door-opening (Additional file 1: Figure S3). In an empty house, the blower door was activated in order to create and maintain a pressure across the building shell, and the flow through the fan measured. Air leakage through the building was calculated and evaluated using the American Society for Testing and Materials method E779-19 [16]. The building leakage is usually defined by ACH50, the amount of air that escapes a building in m^3^/h when exposed to a positive or negative pressure of 50 Pa. In order to get a precise curve for the determination of the ACH50 a minimum of five target building pressures of 5 Pa, 10 Pa, 15 Pa, 20 Pa and 50 Pa was used. The test procedure requires both a depressurization and a pressurization test to be performed, giving a total of 10 measurement points for each final ACH50 value (Additional file 1: Figure S4). During an automated process a large fan in the blower door is either pulling or pushing air in or out of the building while measuring the positive or negative pressure via a pressure gauge. The two measurements are often similar but might differ if openings in the building are working as unidirectional valves. The experiment was carried out six times through a 24-hour cycle; at 02.00 h, 06.00 h, 10.00 h, 14.00 h, 18.00 h and 22.00 h. Each test took approximately 15 min including moving the blower door from one house to the other. The experiment was made on the same day as method B.

#### Method B. Artificial CO2 elevation & decay

Here CO_2_ levels were elevated using compressed gas and the rate of decay used to estimate ACH [12, 24, 25]. All ventilation openings including windows, open eaves and gaps in doors were sealed and all people vacated the room. A 10 mm diameter plastic hose hung from the ceiling, 180 cm from the ground, was connected to a CO_2_ gas canister kept outside the house. CO_2_ was released at a rate of at 500 ml/min for approximately 60 s while CO_2_ levels inside the house was measured from the outside using the software HOBOmobile connecting the CO_2_ data loggers to an Iphone via Bluetooth. In this experiment and all those measuring CO_2_ (Methods B, C and D), CO_2_ data loggers (HOBO^®^ MX CO2 Logger #MX1102, Onset Computer Corporation. 470 MacArthur Blvd., Bourne, MA 02532, USA) were placed inside in the middle of the room, one metre above the ground. When a steady state of CO_2_ of 2000–3000 ppm was reached indoors, the CO_2_ was manually mixed by waving a 30 cm diameter plastic lid for 60 s. While this was done a second person outside the building ensured the safety of the person in the room. After manually mixing the CO_2_, the room was left empty for 20 min for the air to equilibrate before opening all sealed ventilation openings and starting the measurements. Temperature, relative humidity and CO_2_ were measured every 5 s for approximately 15 min (approximately 180 measurements) on both data loggers or until the CO_2_ concentration indoors has declined to values near to that outdoors. The experiment was carried out six times through a 24-h cycle; at 04.00 h, 08.00 h, 12.00 h, 16.00 h, 20.00 h and 00.00 h. Each test took approximately 30 min.

#### Method C. Natural CO_2_ elevation and decay

This experiment was similar to the proceeding one, but with CO_2_ levels raised through human activity, based on previous methodology [12, 25, 26]. All ventilation openings including windows, open eaves and gaps around doors were sealed. Three people walked around inside each house for 20 min or until the expected baseline CO_2_ level of 400–600 ppm was raised to 700–800 ppm, whereupon all people left the house and all sealed ventilation openings opened. Temperature, relative humidity and CO_2_ were measured every 3 min for 8 h (approximately 160 measurements) on both data loggers or until the indoor CO_2_ concentration decreased to values similar to those outdoors, whichever occurred first. The experiment started at 09.00 h and ended at 17.00 h.

#### Method D. Natural CO_2_ rise

This experiment measured the rate at which CO_2_ increased indoors due to human activity and was based on methods developed previously [25, 26]. At the start of the experiment the doors were opened for 30 min to ventilate the house. Two people slept indoors under a LLIN inside the house from 21.00 h to 06.00 h the following morning. Sleepers only left the room under exceptional circumstances. All ventilation openings remained open and temperature, relative humidity and CO_2_ were measured on both data loggers every 3 min for approximately 9 h (approximately 180 measurements). The experiment was carried out daily for 6 days and made on the same day as method C and E.

#### Method E. Hot wire thermo-anemometer

Here a hot wire thermo-anemometer was used to measure indoor air speed (IAS) based on an established methodology [27]. Two hot wire thermo-anemometers (ATP Instrumentation AAVM-8880 Hot Wire USB Logging Thermo-Anemometer, ATP Instrumentation Ltd, Ashby-de-la-Zouch, Leicestershire, United Kingdom) were placed inside the building on a tripod in the middle of the room, one metre above the ground. A CO_2_ data logger, which also record temperature and relative humidity, was placed just below the anemometer. All doors were opened for 30 min, ventilating the house. The internal air volume and surface area of all openings were calculated. Two adult men slept under a LLIN in each house from 21.00 h to 06.00 h. From 22.00 to 06.00 h they were instructed to rest or sleep under a LLIN. All ventilation openings were open. IAS, CO_2_, relative humidity and temperature were measured every 3 min for approximately 9 h (approximately 180 measurements). The experiment was repeated for six consecutive days and made on the same day as method C and D.

### Experiment 2. Comparisons of ACH between housing typologies

Firstly, ventilation was compared in a reference metal-roofed house with closed eaves and badly-fitting doors (MCB) with a similar house (MCB). Secondly the reference metal-roofed house with closed eaves and badly-fitting doors (MCB) was compared with: (1) a house with thatched roof and open eaves and badly-fitting doors (TOB), (2) a metal-roofed house with closed eaves and badly-fitting doors plus eaves tubes (MCBE), (3) a metal-roofed house with closed eaves and screened doors (MCS) and (4) a metal-roofed house with closed eaves, screened doors and screened gable windows (MCSG). All five test methods described under experiment 1 were repeated on each pair of houses for six consecutive days. The indoor mean temperature and humidity was measured over six nights in each house typology.

### Statistical analysis

For method A, mean ACH was determined using the six ACH50 values generated by the blower door software. For method B, C and D, ACH was determined using the natural logarithm CO_2_ concentration plotted over time. The slope of the line represented the ACH. Each test was done 5 times (method B) or 6 times (method C and D) and the mean calculated. For method E, ACH was calculated knowing the IAS (m/s), the volume of the building (m^3^) and the area of openings (m^2^). Six repeated tests were carried out over a 24-h cycle for each house and the mean value calculated. Comparisons between ACH values determined for each house each night were made using paired t tests to adjust for variation between nights. All analyses were performed using IBM SPSS v24 software.

### Ethical considerations

The study was approved by the Gambia Government/Medical Research Council’s joint ethics committee (16th May 2016 and 16th March 2017) and the Department of Biosciences ethics committee, Durham University, UK (13th May 2016 and 29th June 2017).

## Results

### Experiment 1. Ventilation tests

The blower door test showed that there were significant differences in the ACH in two identical houses (Table 1). The standard test, however, requires a pressure of 50 Pa, but due to leakages in the houses it was only possible to achieve a pressure of 25 Pa in the reference metal-roofed house (MCB) or the metal-roofed house with eave tubes (MCBE). For the other house typologies, it was impossible to achieve even the minimum pressure of 5 Pa the test requires. The three methods used for measuring CO_2_ indoors all showed consistent results between the two houses. Both hot wire thermo-anemometers broke during the initial test runs resulting in no data being collected.

**Table 1:** Experiment 1: Ventilation estimated using different methods in two identical houses

Method	Day	Time of recordings	n	House		t	p
				House 1 Mean ACH m^3^/h (95% CI)	House 2 Mean ACH m^3^/h (95% CI)		
Method A: Fan pressurization (blower door test)	1	02.00 h, 06.00 h, 10.00 h, 14.00 h, 18.00 h and 22.00 h	5	71.6 (69.0, 74.3)	85.2 (80.6, 89.9)	− 15.2	<0.001
Method B: Artificial CO_2_ elevation and decay	1	04.00 h, 08.00 h, 12.00 h, 16.00 h, 20.00 h and 00.00 h	5	− 33.2 (− 51.6, − 14.8)	− 29.9 (− 47.2, − 12.7)	− 0.421	0.695
Method C: Natural CO_2_ elevation and decay*	2–7	09.00–17.00 h	6	− 28.0 (− 46.6, − 9.5)	− 20.5 (34.1, − 6.9)	− 1.040	0.346
Method D: Natural CO_2_ rise	2–7	21.00–06.00 h	6	14.8 (12.1, 17.5)	13.2 (12.1, 14.4)	1.465	0.203
Method E: Hot wire thermo-anemometer	2–7	21.00–06.00 h	6	n/a	n/a	–	–

*ACH* air exchange per hour, *CI* confidence intervals

* First 15 min after human subjects have entered the house

### Experiment 2. Comparisons of ACH between housing typologies

ACH was higher in houses with thatched-roofs and open eaves (TOB), and metal-roofed houses with screened doors (MCS), and metal-roofed houses with screened doors and screened windows in the gable ends (MCSG) compared with the reference metal-roofed house (MCB) assessed using three different methods for measuring changes in indoor CO_2_ levels (Table 2). There was, however, no difference in ACH between metal-roofed houses with eaves (MCBE) and the reference metal-roof house (MCB). All results in experiment 2 came from the three CO_2_ methods, as both the blower door test and the hot wire thermos-anemometers failed. A nightly summary of these findings is shown in Additional file 2: Table S1.

**Table 2:** Experiment 2: Comparison of ventilation using CO_2_ data loggers between different typologies of housing

Typology comparison	Method														
	Method B: Artificial CO_2_ elevation and decay (n = 5)					Method C: Natural CO_2_ elevation and decay* (n = 6)					Method D: Natural CO_2_ rise (n = 6)				
	Control house, mean ACH m^3^/h (95% CI)	Test house, mean ACH m^3^/h (95% CI)	Difference, mean ACH m^3^/h (95% CI)	t	p	Control house, mean ACH m^3^/h (95% CI)	Test House, mean ACH m^3^/h (95% CI)	Difference, mean ACH m^3^/h (95% CI)	t	p	Control house, mean ACH m^3^/h (95% CI)	Test house, mean ACH m^3^/h (95% CI)	Difference, mean ACH m^3^/h (95% CI)	t	p
	− 38.1 (− 42.2, − 34.0)	− 132.2 (− 182.6, − 81.8)	94.1 (43.0, 145.1)	5.112	0.007	− 11.3 (− 23.4, − 0.8)	− 31.6 (− 44.5, 18.7)	20.3 (4.4, 36.2)	3.280	0.022	13.2 (9.4, 17.0)	0.1 (− 2.3− 2.4)	13.1 (11.3, 15.0)	18.071	< 0.001
	− 22.0 (− 33.0, − 13.0)	− 23.7 (− 34.2, − 13.1)	1.6 (− 11.3, 14.6)	0.347	0.746	− 12.4 (− 16.9, − 7.8)	− 8.0 (− 15.7, − 0.3)	− 4.3 (− 13.3, 4.7)	− 1.236	0.271	14.1 (10.2, 18.0)	11.1 (7.2, 14.9)	3.1 (− 0.2, 6.4)	2.396	0.062
	− 12.2 (− 33.1, 8.6)	− 120.2 (− 156.9, − 83.6)	108.0 (55.2, 160.8)	5.681	0.005	− 7.6 (− 12.7, − 2.6)	− 42.2 (− 55.8, − 28.5)	34.5 (20.7, 48.4)	6.405	0.001	14.7 (12.9, 16.6)	6.1 (4.2, 8.1)	8.6 (6.2, 11.0)	9.317	< 0.001
	− 24.2 (− 42.6, − 5.8)	− 151.5 (− 202.1, − 100.9)	127.3 (74.6, 180.1)	6.699	0.003	− 5.8 (− 15.0, 3.4)	− 36.2 (− 49.0, − 23.4)	30.4 (13.3, 47.6)	4.556	0.006	13.3 (12.1, 14.5)	− 1.3 (− 4.2, 1.6)	14.6 (11.6− 17.6)	12.349	< 0.001

*ACH* air exchange per hour, *CI*  confidence intervals

* First 15 min after human subjects have entered the house

**Fig. 2:**
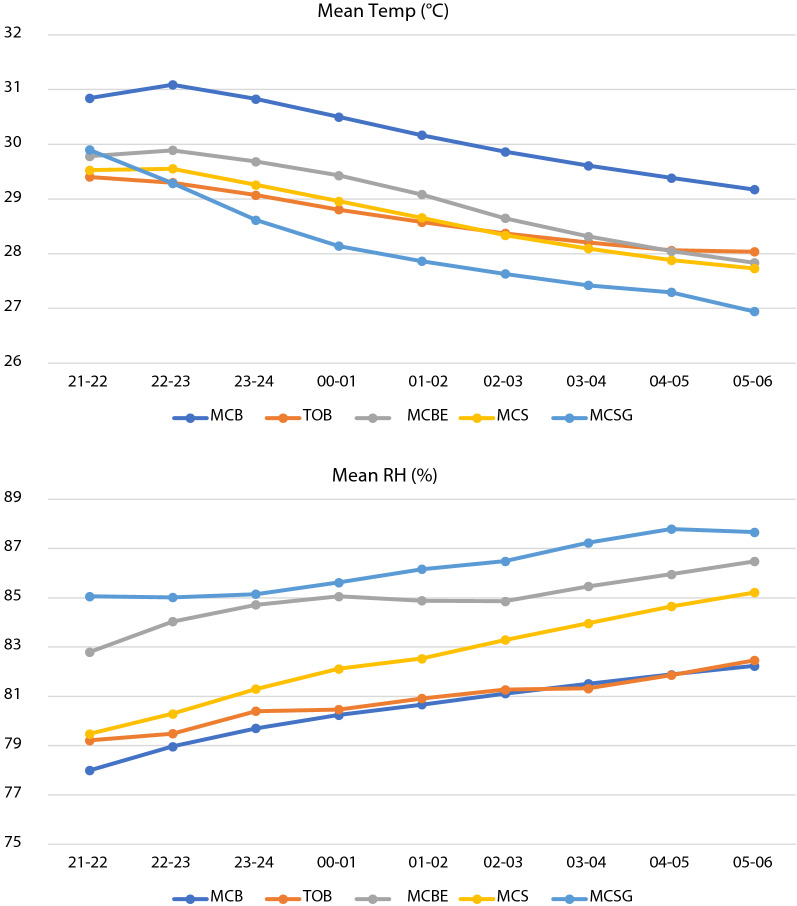
Indoor temperature and relative humidity

All houses showed similar patterns with highest temperature in the evening dropping gradually over night (Fig. 2). Relative humidity showed the opposite pattern with raising values over during the night. Metal-roof houses with eaves tubes (MCBE) and metal-roof houses with screened doors (MCS) were cooler than the reference MCB house in the periods before and after midnight (Table 3). The coolest houses were those with thatch roof and open eaves (TOB) or metal-roof houses with screened doors and gable windows (MCSG), and were 1.5 °C cooler than the reference house (MSB).

**Table 3:** Temperature and humidity measured indoors before and after midnight in experiment D

Typology comparison	Temperature (^o^C) (n = 6)					Relative Humidity (%) (n = 6)				
	Control house, mean temp (^o^C)	Test house, mean temp (^o^C)	Difference, mean temp (^o^C)	t	p	Control house, mean RH %	Test house, mean RH %	Difference, mean RH %	t	p
21.00–24.00 h										
	31.7 (30.7–32.6)	31.6 (30.7–32.5)	0.1	1.029	0.351	72.2 (68.2–76.2)	78.4 (75.6–81.3)	− 6.2	− 3.001	0.030
	30.7 (29.6–31.9)	29.3 (28.2–30.3)	1.5	11.099	< 0.001	81.4 (79.2–83.6)	83.8 (82.2–85.4)	− 2.4	− 6.316	0.01
	30.2 (28.5–32.0)	29.8 (27.7–31.2)	0.8	13.458	< 0.001	81.4 (79.6–85.3)	83.8 (75.5–85.1)	− 2.4	− 6.316	0.001
	30.2 (28.5–32.0)	29.6 (27.7–31.2)	0.8	13.458	< 0.001	82.5 (79.6–85.3)	80.3 (75.5–85.2)	2.2	2.572	0.050
	30.8 (30.1–31.4)	29.3 (28.6–30.0)	1.5	17.034	< 0.001	85.6 (84.9–86.3)	85.1 (83.8–86.4)	0.5	1.349	0.235
00.00–06.00 h										
	30.7 (30.0–31.4)	30.5 (29.8–31.2)	0.2	11.918	< 0.001	76.7 (74.5–78.9)	74.8 (72.2–77.3)	1.9	8.784	<0.001
	29.8 (28.9–30.8)	28.4 (27.5–29.2)	1.5	16.403	< 0.001	80.1 (77.2–82.9)	81.4 (77.6–85.2)	− 1.3	− 2.618	0.047
	29.4 (28.2–30.5)	28.6 (27.4–29.7)	0.8	13.093	< 0.001	81.4 (79.2–83.6)	85.4 (83.8–87.1)	− 4.0	− 6.985	0.001
	29.2 (27.8–30.5)	28.3 (27.0–29.6)	0.9	15.144	< 0.001	85.3 (83.3–87.2)	83.6 (80.2–87.0)	1.7	2.747	0.040
	29.2 (28.5–29.8)	27.6 (26.9–28.3)	1.6	36.3	< 0.001	87.9 (87.0–88.7)	86.8 (85.5–88.1)	1.1	2.203	0.079

## Discussion

The findings demonstrate that ventilation can be measured in a range of housing typologies common in rural sub-Saharan Africa using a variety of different methods. Although the blower door is an industrial standard used to evaluate how airtight a building is, buildings with open eaves or large areas of screening were too leaky to make recordings. The blower door is designed to measure building leakage through small cracks and openings, but our buildings, with high levels of leakage, could not reach the minimum pressure required of 25 Pa. A larger and more powerful version, however, might have been able to work. All methods that measured rising or falling levels of CO_2_ using data loggers to estimate ventilation in Gambian houses performed well. Raising CO_2_ levels indoors using gas released from a CO_2_ cylinder, was easy to use, cheap (< $1/test) and could easily be adapted to use for testing of ACH in around 30 min. It should be recognized, however, that high concentrations of CO_2_ are potentially lethal and that the house should be well ventilated before allowing people to return to a house after a test has been made. Raising CO_2_ concentrations naturally using people indoors, was simple to use and worked well. It took 20 min or less before a sufficiently high concentration of CO_2_ was obtained. With this method there is no danger of fatalities as CO_2_ concentrations are relatively low and measurement needs only to be taken for 15 min to obtain a reading. Measuring the natural rate of CO_2_ increase indoors, was simple to perform and worked well in poorly ventilated buildings, but failed to increase in well-ventilated structures. The hot wire thermo-anemometers performed badly in the field. Although staff were well instructed in handling the equipment both instruments broke after a few days use. The fragility of the instruments makes it very difficult to recommend hot wire thermo-anemometers for evaluating AIS under field conditions. Hot-wire thermo-anemometers can be used successfully to evaluate airflow in very controlled surroundings like a ventilation duct, but they are of little use inside African houses, at least in this study. Other brands of hot-wire thermo-anemometers could potentially be more reliable, but they are all constructed from fragile components.

All measurements of ventilation varied within and between nights as they were influenced by the external weather, not least changes in wind intensity and direction. Nonetheless, consistent differences in ventilation between different housing typologies was demonstrated. The three methods measuring CO_2_ produced broadly similar results and are likely to be a reasonable measure of ventilation in rural structures. The initial validation comparing two identical metal-roofed houses with closed eaves and badly-fitting doors (MCB–MCB) had similar values for ACH when tested using the three different CO_2_ methods. The MCB–TOB comparison shows that traditional houses with open eaves and thatch roofs are much better ventilated than metal-roof houses with closed eaves. This finding is concerning since the traditional thatch-roofed houses are being converted to metal-roofed ones across many parts of sub-Saharan Africa [2]. The consequences of the reduced ventilation in metal-roofed houses are that the houses will be hotter and more uncomfortable [8], and thus people are less likely to sleep under a LLIN and therefore be at increased risk of malaria. On the other hand, a high proportion of metal-roofed houses in a settlement may increase the population mortality of mosquitoes resting indoors, reducing the risk of malaria in a community [6]. Method D, designed to measure the natural rise in CO_2_, failed to increase in the TOB house illustrating that ventilation in this typology is markedly greater than that experienced in the metal-roof house (Table 3).

Eave tubes are one of the promising new interventions being developed for malaria control in sub-Saharan Africa [28]. The comparison between metal-roofed houses without and with eave tubes (MCB-MCBE) suggest that eave tubes do not increase indoor ventilation as assessed using CO_2_, although houses with eave tubes were slightly cooler than houses without, suggesting any impact is small. This finding is not surprising since the cross-sectional area of the tubes (0.06 m^2^) is very small compared to the volume of the building (38 m^2^), equivalent to two screened windows, measuring 30 cm × 20 cm on each side of the building. In terms of cross-ventilation this is very little. Although host odours from inside the house may be released through the tubes, the eave tubes do not increase ventilation, leaving indoor comfort unchanged. On the other hand, the MCB-MCS comparison shows that large screened doors will increase ventilation and therefore indoor comfort, and this can be further increased by inserting screened windows in the gable ends of buildings, as seen in the MCB-MCSG comparison. Indeed, in the typology with screened doors and windows, using the natural method for producing CO_2_ (method D) we could not raise the CO_2_ concentration indoors in the MSCG house, indicating that this house is in ventilation balance comparable to the traditional thatch-roof houses.

In this study the metal-roofed house with closed eaves was markedly hotter at night than the traditional thatch houses, in agreement with previous studies [8]. Nonetheless, the metal-roofed house could be made cooler by adding screened doors and windows to the house.

In this study, methods to measure ventilation have been evaluated. Knowledge on ventilation in a house could be used to keep the occupants cool and more likely to sleep under a bed net. Another important consideration is to better understand how air moves out of these houses in a more detailed way, as these currents of air disseminate human odours and enable mosquitoes to locate human hosts [29]. Airflow, and the shape of the host odour plume leaving the house can be modelled using Computer Fluid Dynamics (CFD) software [30]. The results of the current experiment will be used to evaluate a future CFD modelling study designed to improve airflow in buildings to keep the occupants comfortable, whilst keeping out mosquitoes.

## Conclusion

This pilot study has identified simple, practical and accurate methods for measuring ventilation in rural African houses. Indoor ventilation is essential in hot and humid environments if people are going to be comfortable and use a bed net at night. Indoor climate is normally only evaluated looking at temperature and humidity leaving out ventilation, the third major component in indoor thermal comfort [19]. This study found that the simplest method for reliably evaluating natural ventilation of a building was to use CO_2_ data loggers to measure natural concentrations of CO_2_ decay. In houses that are well ventilated the best way to do this is use artificial sources of CO_2_ to rapidly raise indoor concentrations, taking care that no one remains inside the house during these measurements.

Roll Back Malaria, United Nations (UN) Development Program and UN Habitat’s Housing and Malaria consensus statement [24] has asked ‘what architectural features are protective’ against malaria? One of the answers is “climatically comfortable houses that allows people to use a LLIN”. This study demonstrates that the climatic performance of different house typologies differs a lot in rural Gambia. Traditional houses with open eaves and thatch roofs are much better ventilated than the more modern and now typical metal-roof houses with closed eaves. This could be a major problem as the transformation from thatch to metal is happening all over Africa at a very rapid pace. A metal-roofed house can, however, be made as well ventilated as a thatched-roofed house by allowing airflow through the house by screening the doors and adding a screened window directly under the roof. Better ventilation and higher airflow increased thermal comfort makes it more likely that people will sleep under a LLIN, helping to improve malaria control in hot and humid parts of sub-Saharan Africa.

## Supplementary information

**Additional file 1: Figure S1.** Aerial Photo. Wali Kunda Field Station (13° 34.440′ N, 14° 55.471′ W). North up. **Figure S2.** House typologies. 1) Metal roof, closed eaves, badly-fitted solid doors, MCB, 2) thatched-roofed house with closed eaves and badly-fitting doors, TOB, 3) Metal roof, closed eaves, badly-fitted solid doors and eave tubes, MCBE, 4) Metal roof, closed eaves, well-fitted ventilated screened door, MCS and 5) Metal roof, closed eaves, well-fitted ventilated screened doors & ventilated gable ends, MCSG (designed by JK). **Figure S3.** Photos. 1) Aerial photo, 2) experimental houses seen from north, 3) interior of house with BlowerDoor and eave tubes, 4) exterior with BlowerDoor inserted, 5) custom-made eave tubes 6) roof being replaced, 7) close-up of screened door and 8) ventilated window under roof. **Figure S4.** BlowerDoor test. Example on output from automated test. **Figure S5.** ACH estimated using CO2 data loggers. Test A, Test B and Test C.

**Additional file 2: Table S1.** Summary data of air exchange with different typologies of houses.

## Data Availability

The datasets used and/or analysed during the current study are available from the corresponding author on reasonable request.

## Abbreviations

ACH: Air exchange pr. hour (number)
IAS: Indoor air speed (m/s)
