# Supplementary material for: Measuring ventilation in different typologies of rural Gambian houses: a pilot experimental study
Source: Malar J. 2020 Jul 31;19:273. doi: 10.1186/s12936-020-03327-0 (PMC7393878; doi:10.1186/s12936-020-03327-0)
Supplement: Supplementary file 1 — Additional file 1: Figure S1. Aerial Photo. Wali Kunda Field Station (13° 34.440′ N, 14° 55.471′ W). North up. Figure S2. House typologies. 1) Metal roof, closed eaves, badly-fitted solid doors, MCB, 2) thatched-roofed house with closed eaves and badly-fitting doors, TOB, 3) Metal roof, closed eaves, badly-fitted solid doors and eave tubes, MCBE, 4) Metal roof, closed eaves, well-fitted ventilated screened door, MCS and 5) Metal roof, closed eaves, well-fitted ventilated screened doors & ventilated gable ends, MCSG (designed by JK). Figure S3. Photos. 1) Aerial photo, 2) experimental houses seen from north, 3) interior of house with BlowerDoor and eave tubes, 4) exterior with BlowerDoor inserted, 5) custom-made eave tubes 6) roof being replaced, 7) close-up of screened door and 8) ventilated window under roof. Figure S4. BlowerDoor test. Example on output from automated test. Figure S5. ACH estimated using CO2 data loggers. Test A, Test B and Test C. [file 12936_2020_3327_MOESM1_ESM.pdf]

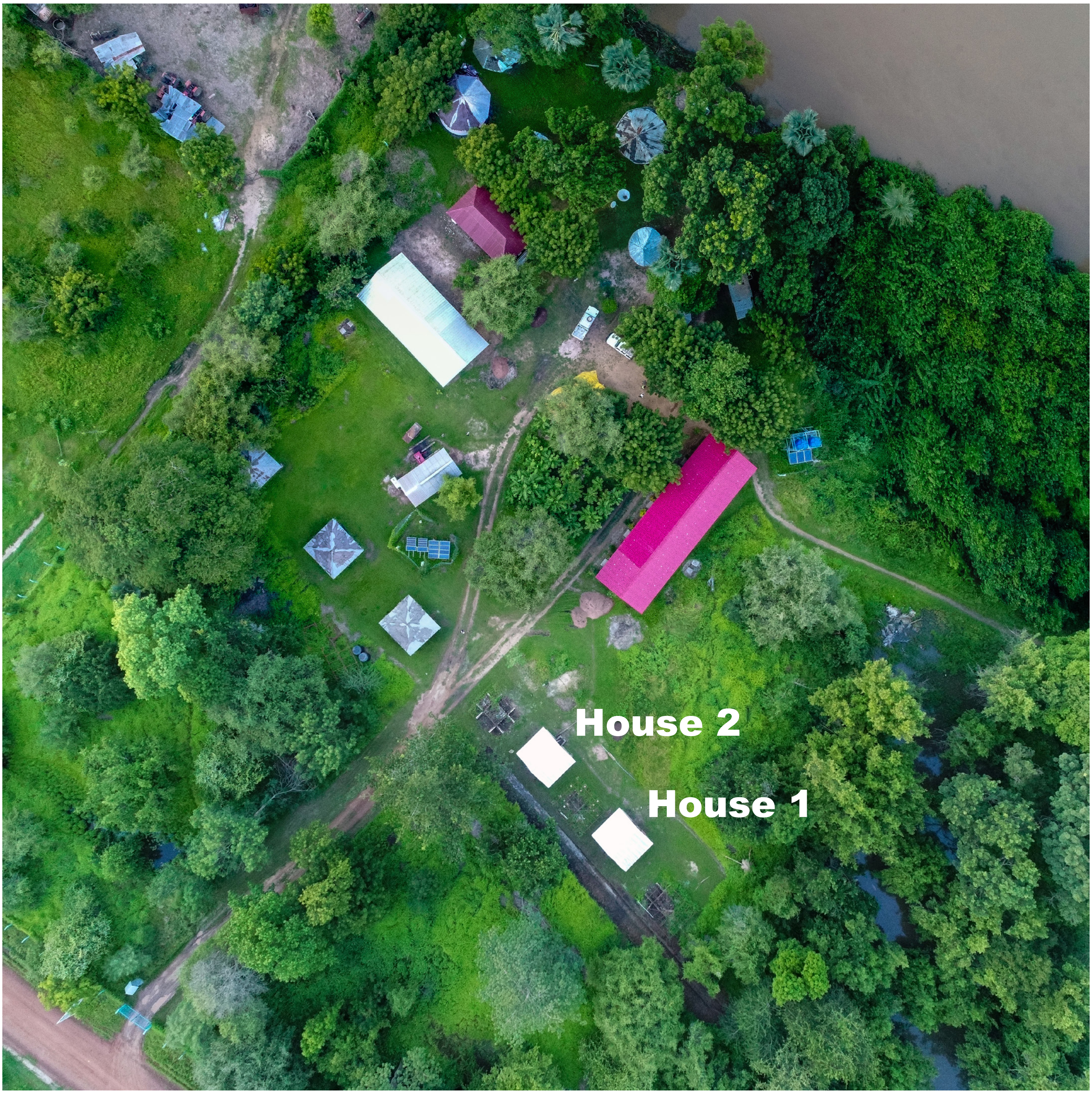
House 2
House 1

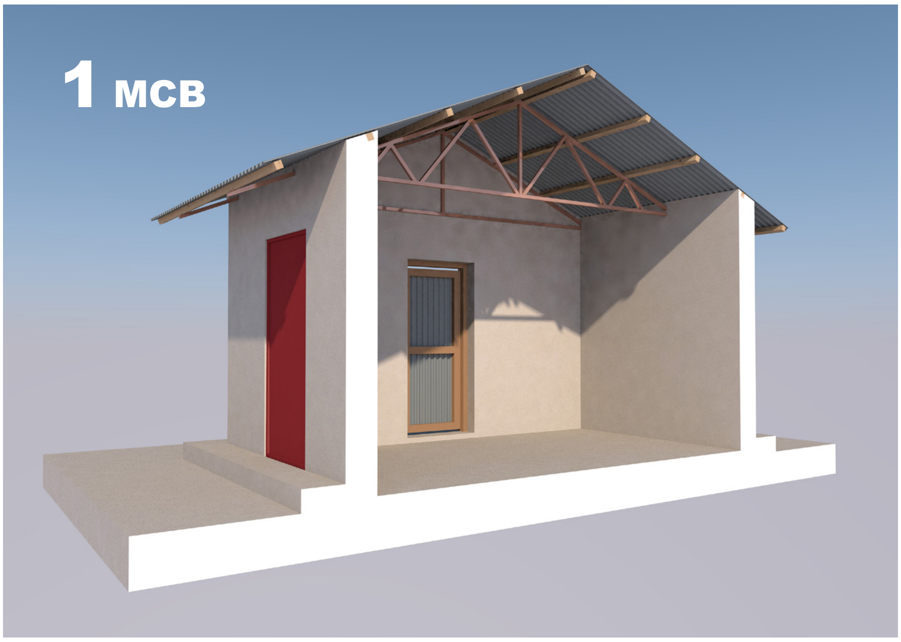
1 MCB

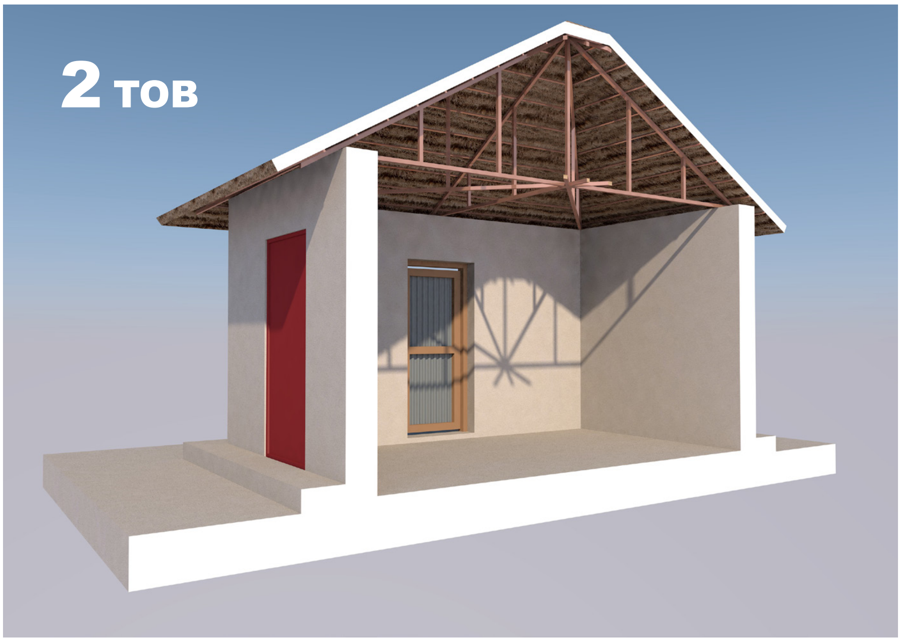
2 TOB

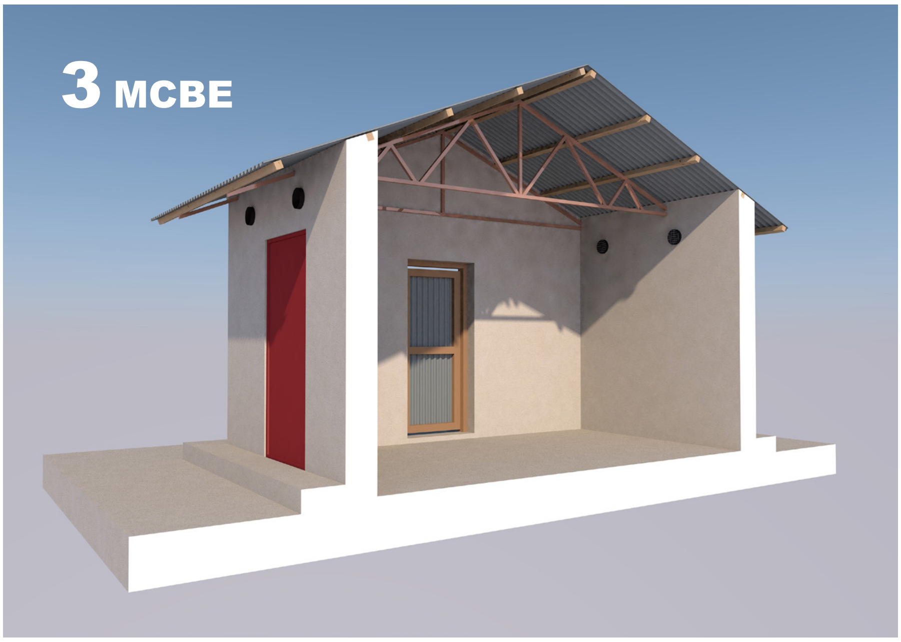
3 MCBE

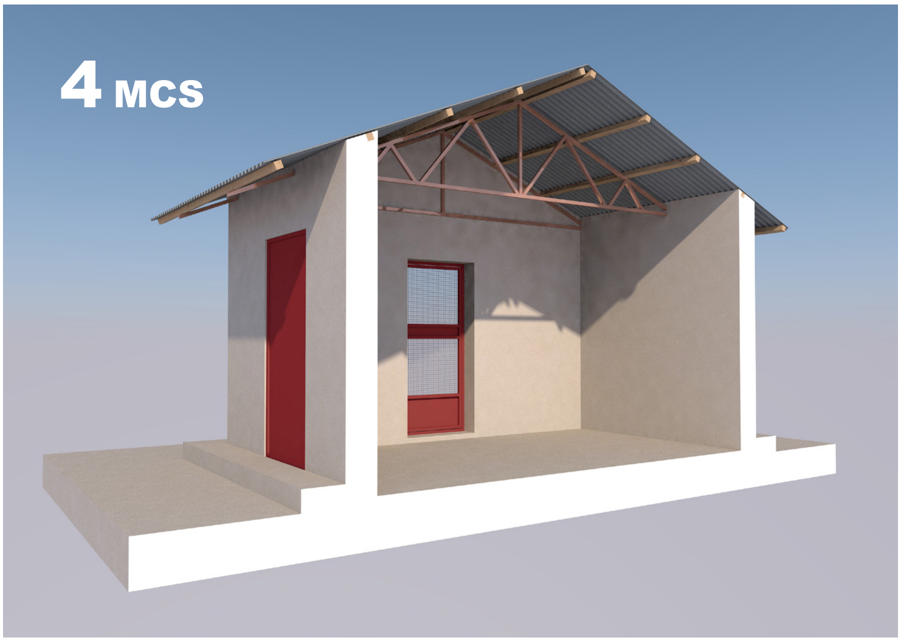
4 MCS

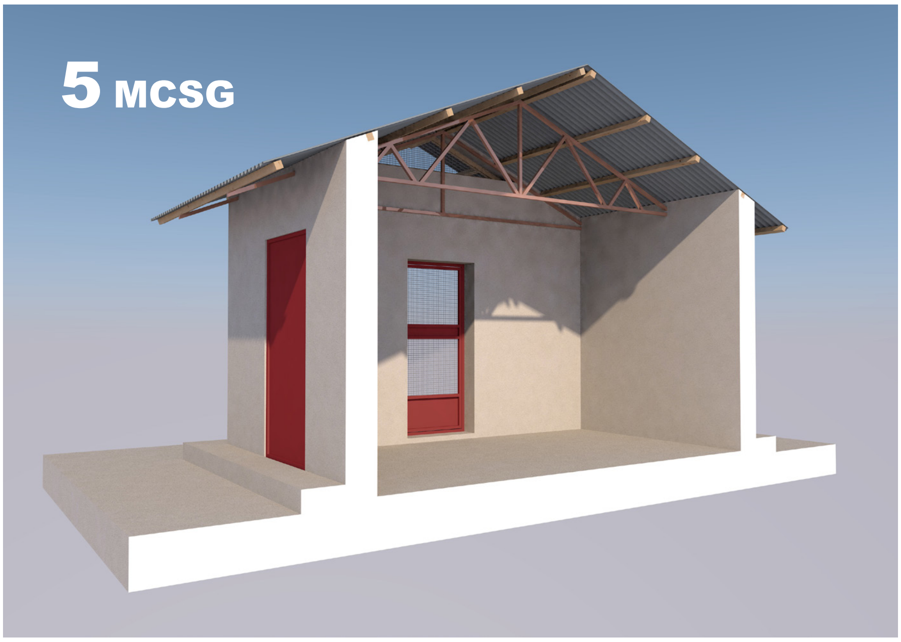
5 MCSG

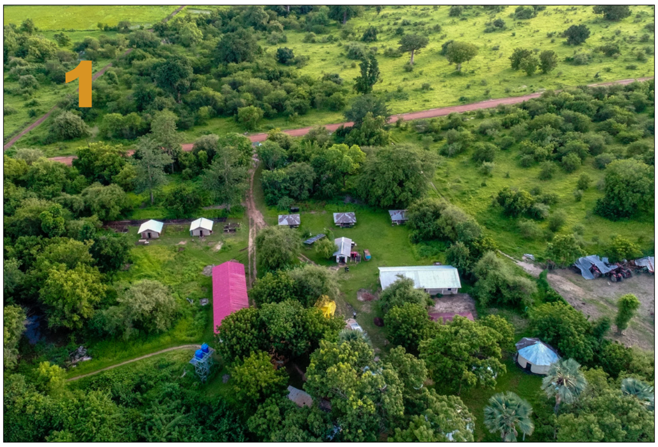
1
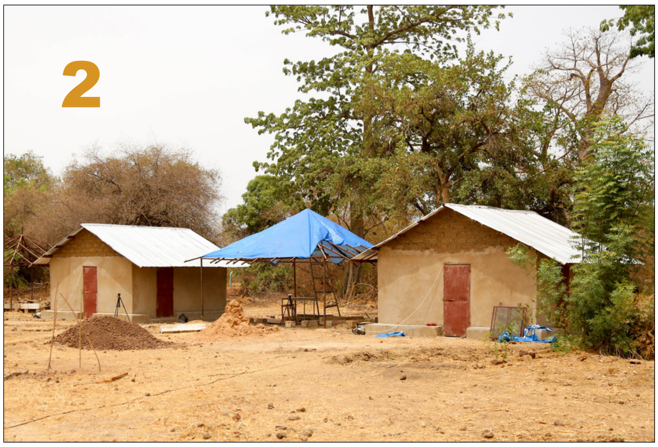
2
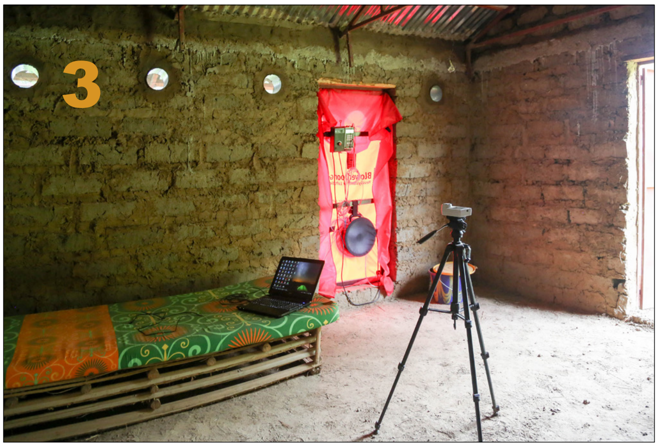
3
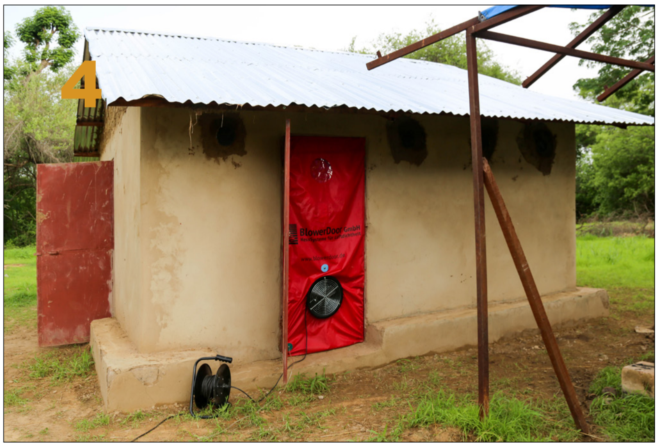
4
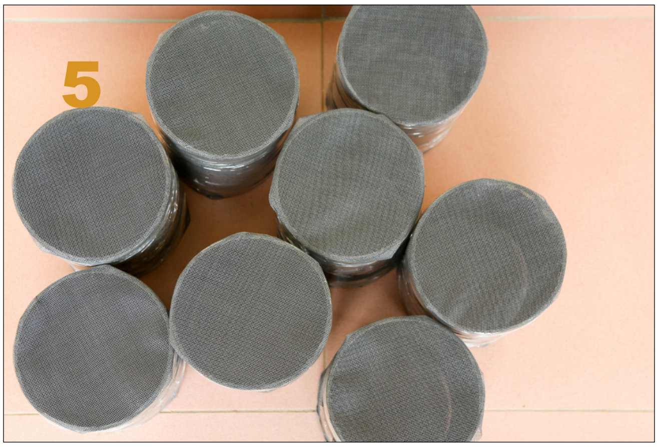
5
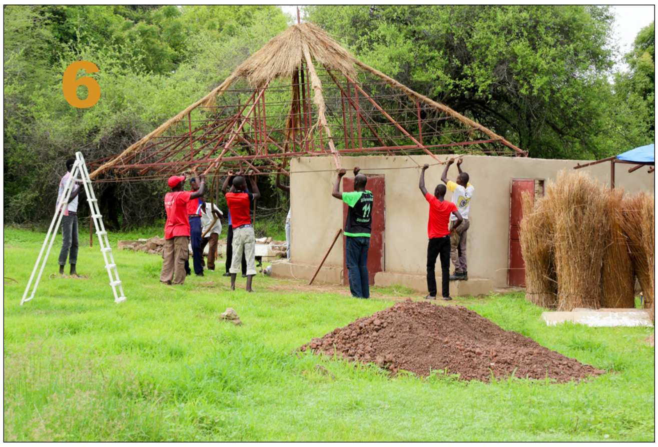
6
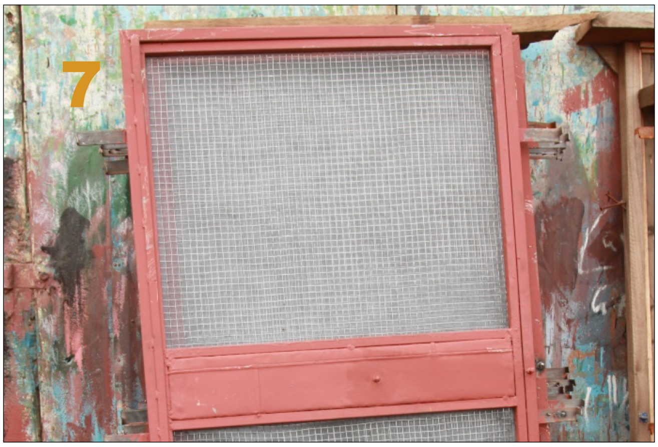
7
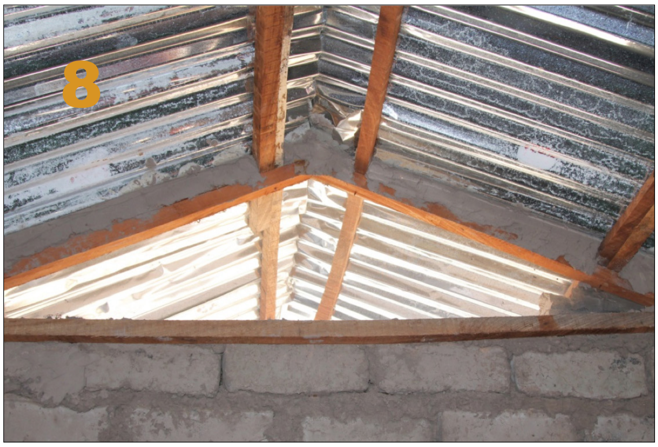
8

BUILDING LEAKAGE TEST

Date of Test: 01-07-2017   Test File: 170701-HUT 1 Pressurization

Technician: Ingvartsen Arhitects

Project Number:

Customer: Endgame

Building Address: HUT 1
Wali Kunda

Phone:

Fax:

	Depressurization	Pressurization	Average
**Test Results at 50 Pascals:**			
m³/h (Airflow)	3220 (+/- 15.0 %)	3533 (+/- 18.6 %)	3377 (+/- 12.1 %)
ACH50	65.19	71.53	68.36
m³/(h·m² Floor Area)	182.9666	200.7656	191.8661
m³/(h·m² Surface Area)	46.0688	50.5504	48.3096
**Leakage Areas:**			
Canadian EqLA @ 10 Pa (cm²)	1492.7 (+/- 5.5 %)	1567.3 (+/- 6.8 %)	1530.0 (+/- 4.4 %)
cm²/m² Surface Area	21.35	22.42	21.89
LBL ELA @ 4 Pa (cm²)	874.5 (+/- 15.5 %)	895.5 (+/- 19.2 %)	885.0 (+/- 12.4 %)
cm²/m² Surface Area	12.51	12.81	12.66
**Building Leakage Curve:**			
Flow Coefficient (C)	380.8 (+/- 31.7 %)	375.4 (+/- 39.2 %)	378.1 (+/- 25.2 %)
Exponent (n)	0.546 (+/- 0.118)	0.573 (+/- 0.146)	0.559 (+/- 0.094)
Correlation Coefficient	0.99986	0.99980	

Test Standard: E779-10

Test Mode: Depressurization and Pressurization

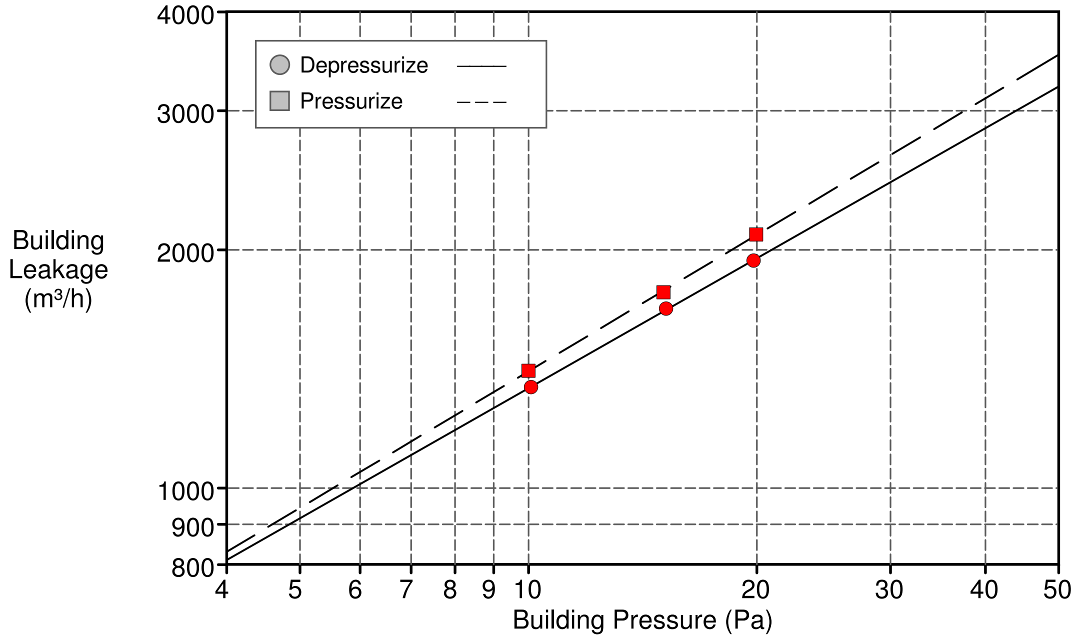

Test B. ACH measured using C02 Decay Method. Raised Concentrations

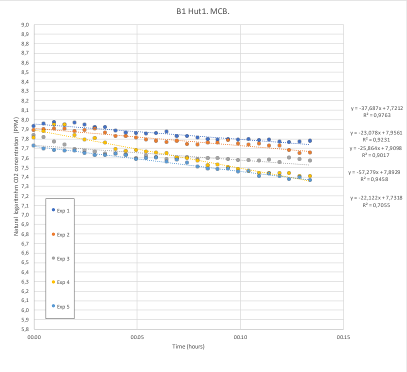

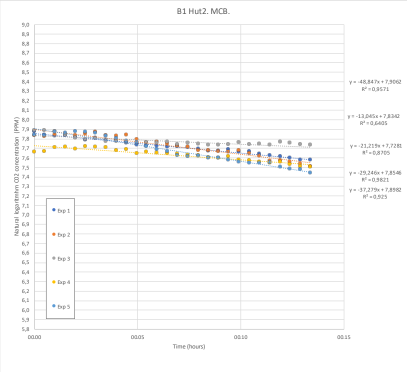

ACH (Air exchange hour)

B1 Hut1. MCB	B1 Hut2. MCB
-37,687	-48,847
-23,078	-13,045
-25,864	-21,219
-57,279	-29,246
-22,122	-37,279
-33,206	-29,927
	90%

MCB MCB

Mean hut 1		-33,2060
95%	Lower Bound	-51,6116
Confidence	Upper Bound	-14,8004

Mean hut 2		-29,9272
95%	Lower Bound	-47,1916
Confidence	Upper Bound	-12,6628

Paired Samples Test

		Paired Differences							
					Difference				
		Mean	Std. Deviation	Std. Error Mean	Lower	Upper	t	df	Sig. (2-tailed)
Pair 1	VAR00001 - VAR00002	-3,27880	17,38256	7,77372	-24,86210	18,30450	-,422	4	,695

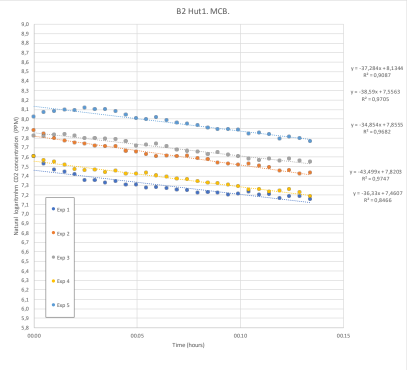

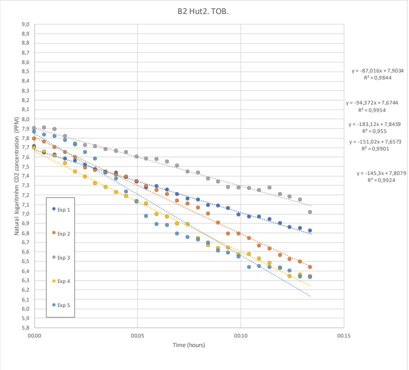

ACH (Air exchange hour)

B2 Hut1. MCB	B2 Hut2. TOB
-37,284	-87,016
-38,590	-94,372
-34,854	-183,120
-43,499	-151,020
-36,330	-145,300
-38,111	-132,166
	347%

MCB TOB

Mean hut 1		-38,1114
95%	Lower Bound	-42,2166
Confidence	Upper Bound	-34,0062

Mean hut 2		-132,1656
95%	Lower Bound	-182,5686
Confidence	Upper Bound	-81,7626

Paired Samples Test

		Paired Differences							
					Difference				
		Mean	Std. Deviation	Std. Error Mean	Lower	Upper	t	df	Sig. (2-tailed)
Pair 1	VAR00001 - VAR00002	94,05420	41,14600	18,40105	42,96469	145,14371	5,111	4	,007

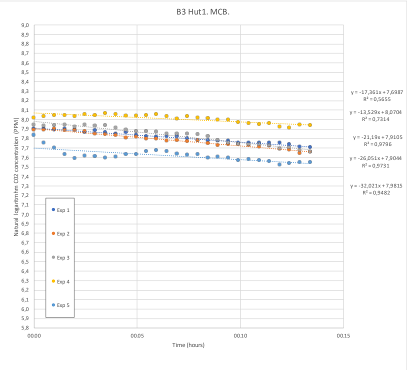

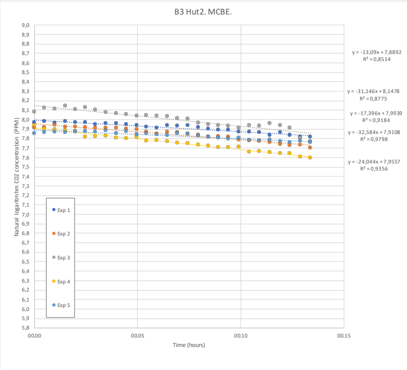

ACH (Air exchange hour)

B3 Hut1. MCB	B3 Hut2. MCBE
-17,361	-13,090
-13,529	-31,146
-21,190	-17,396
-26,051	-32,584
-32,021	-24,044
-22,030	-23,652
	107%

MCB MCBE

Mean hut 1		-22,0304
95%	Lower Bound	-31,0434
Confidence	Upper Bound	-13,0174

Mean hut 2		-23,6520
95%	Lower Bound	-34,1657
Confidence	Upper Bound	-13,1383

Paired Samples Test

		Paired Differences					
					Difference		
		Mean	Std. Deviation	Std. Error Mean	Lower	Upper	t
Pair 1	VAR00001 - VAR00002	1,62160	10,44221	4,66990	-11,34412	14,58732	,347

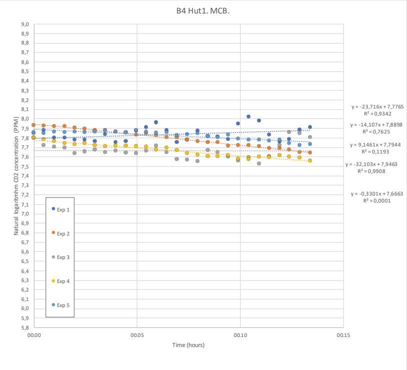

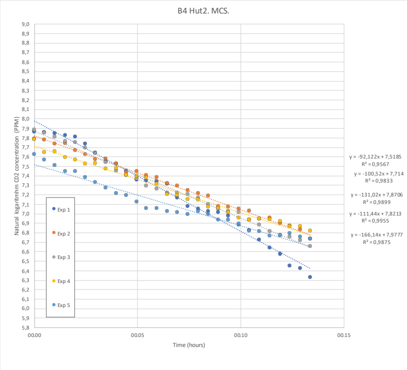

ACH (Air exchange hour)

B4 Hut1. MCB	B4 Hut2. MCS
-23,716	-92,122
-14,107	-100,520
9,146	-131,020
-32,103	-111,440
-0,330	-166,140
-12,222	-120,248
	984%

MCB MCS

Mean hut 1		-12,2220
95%	Lower Bound	-33,0817
Confidence	Upper Bound	8,6377

Mean hut 2		-120,2484
95%	Lower Bound	-156,8720
Confidence	Upper Bound	-83,6248

Paired Samples Test

		Paired Differences					
					Difference		
		Mean	Std. Deviation	Std. Error Mean	Lower	Upper	t
Pair 1	VAR00001 - VAR00002	108,02640	42,52028	19,01565	55,23051	160,82229	5,681

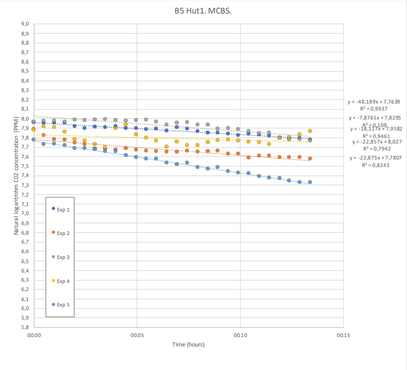

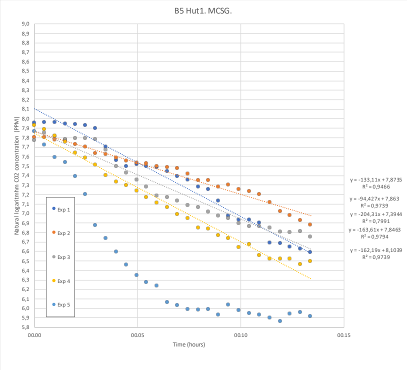

ACH (Air exchange hour)

B5 Hut1. MCB	B5 Hut2. MCSG
-48,189	-133,110
-7,876	-94,427
-18,117	-204,310
-22,857	-163,610
-23,875	-162,190
-24,183	-151,529
	627%

MCB MCSG

Mean hut 1		-24,1828
95%	Lower Bound	-42,6086
Confidence	Upper Bound	-5,7570

Mean hut 2		-151,5294
95%	Lower Bound	-202,1390
Confidence	Upper Bound	-100,9198

Paired Samples Test

		Paired Differences					
					Difference		
		Mean	Std. Deviation	Std. Error Mean	Lower	Upper	t
Pair 1	VAR00001 - VAR00002	127,34660	42,50632	19,00940	74,56803	180,12517	6,699

Test C. ACH measured using CO_2 decay method. Natural concentrations (First 15 minuttes)

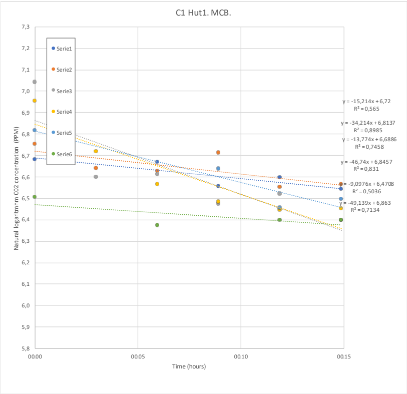

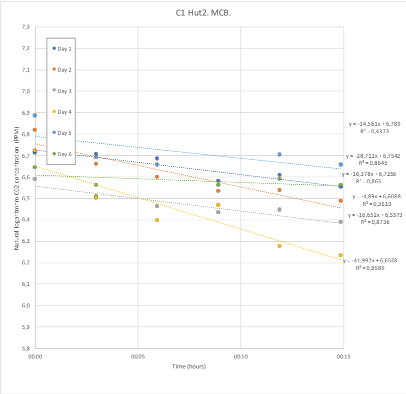

ACH (Air exchange hour)

C1 Hut1. MCB	C1 Hut1. MCB
-15,214	-14,561
-34,214	-28,712
-13,774	-16,378
-46,740	-4,890
-9,098	-16,652
-49,139	-41,992
-28,030	-20,531
	73%

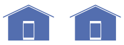

Paired Samples Statistics

		Mean	N	Std. Deviation	Std. Error Mean
Pair 1	VAR00002	-28,0298	6	17,66239	7,21064
	VAR00003	-20,5308	6	12,96209	5,29175

Paired Samples Test

		Paired Differences					t	df	Sig. (2-tailed)
		Mean	Std. Deviation	Std. Error Mean	the Difference Lower	the Difference Upper			
Pair 1	VAR00002 - VAR00003	-7,49900	17,66145	7,21026	-26,03356	11,03556	-1,040	5	,346

No difference

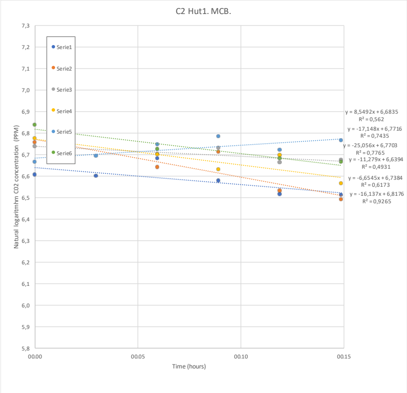

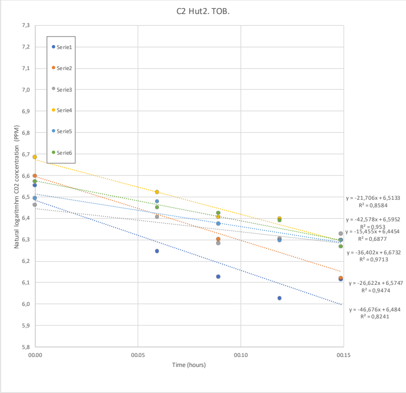

ACH (Air exchange hour)

C2 Hut1. MCB	C2 Hut2. TOB
8,549	-21,706
-17,148	-42,578
-25,056	-15,455
-11,279	-36,402
-6,654	-26,622
-16,137	-46,676
-11,287	-31,573
	280%

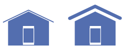

Paired Samples Statistics

		Mean	N	Std. Deviation	Std. Error Mean
Pair 1	VAR00002	-11,2875	6	11,50992	4,69890
	VAR00003	-31,5732	6	12,28161	5,01395

Paired Samples Test

		Paired Differences					t	df	Sig. (2-tailed)
		Mean	Std. Deviation	Std. Error Mean	the Difference Lower	the Difference Upper			
Pair 1	VAR00002 - VAR00003	20,28567	15,15055	6,18519	4,38614	36,18520	3,280	5	,022

statistically different

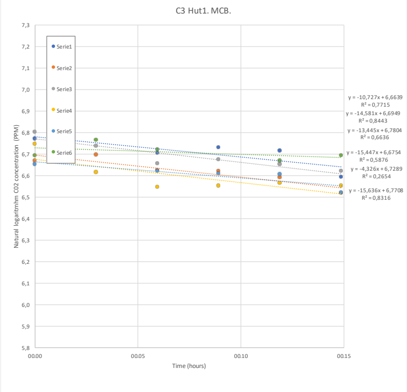

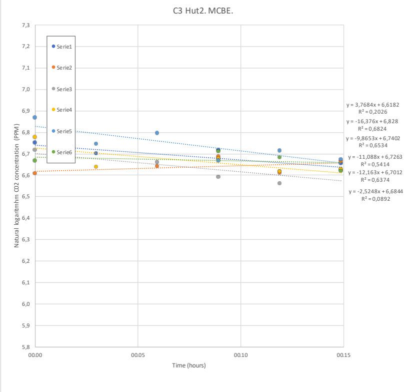

ACH (Air exchange hour)

C3 Hut1. MCB	C3 Hut2. MCBE
-10,727	3,768
-14,581	-16,376
-13,445	-9,865
-15,447	-11,088
-4,326	-12,163
-15,636	-2,525
-12,360	-8,041
	65%

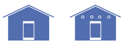

Paired Samples Statistics

		Mean	N	Std. Deviation	Std. Error Mean
Pair 1	VAR00002	-12,3603	6	4,32628	1,76620
	VAR00003	-8,0415	6	7,33421	2,99418

Paired Samples Test

		Paired Differences					t	df	Sig. (2-tailed)
		Mean	Std. Deviation	Std. Error Mean	the Difference Lower	the Difference Upper			
Pair 1	VAR00002 - VAR00003	-4,31883	8,56209	3,49546	-13,30420	4,66653	-1,236	5	,272

No difference

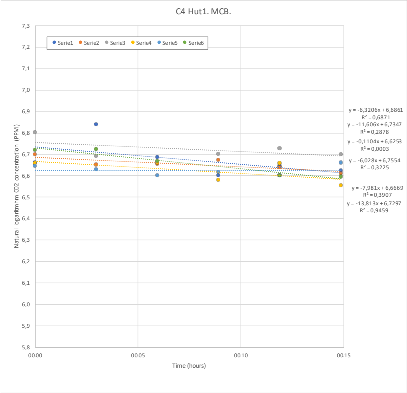

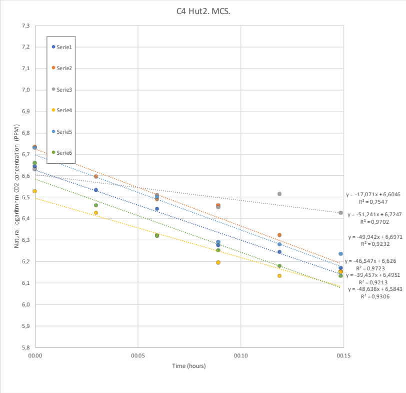

ACH (Air exchange hour)

C4 Hut1. MCB	C4 Hut2. MCS
-6,321	-17,071
-11,606	-51,241
-0,110	-49,942
-6,028	-46,547
-7,981	-39,457
-13,814	-48,638
-7,643	-42,149
	551%

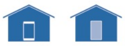

Paired Samples Statistics

		Mean	N	Std. Deviation	Std. Error Mean
Pair 1	VAR00002	-7,6433	6	4,79589	1,95791
	VAR00003	-42,1493	6	12,96918	5,29464

Paired Samples Test

		Paired Differences					t	df	Sig. (2-tailed)
		Mean	Std. Deviation	Std. Error Mean	the Difference Lower	the Difference Upper			
Pair 1	VAR00002 - VAR00003	34,50600	13,19832	5,38819	20,65521	48,35679	6,404	5	,001

statistically significant

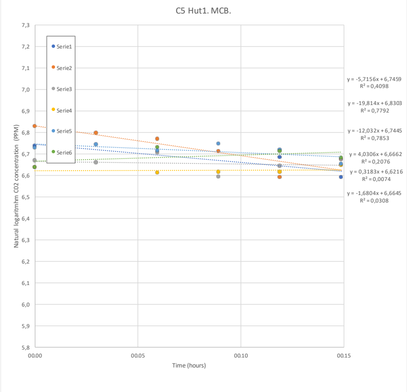

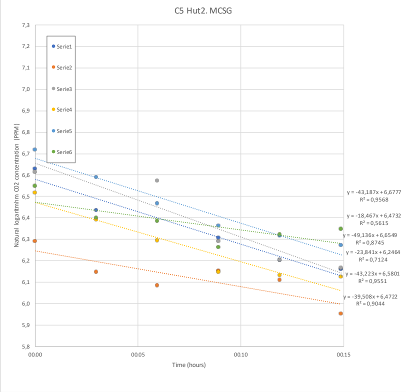

ACH (Air exchange hour)

C5 Hut1. MCB	C5 Hut2. MCSG
-5,715	-43,187
-19,814	-18,467
-12,032	-49,136
4,030	-23,841
0,318	-43,223
-1,680	-39,508
-5,816	-36,227
	623%

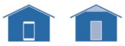

Paired Samples Statistics

		Mean	N	Std. Deviation	Std. Error Mean
Pair 1	VAR00002	-5,8155	6	8,78941	3,58826
	VAR00003	-36,2270	6	12,19527	4,97870

Paired Samples Test

		Paired Differences					t	df	Sig. (2-tailed)
		Mean	Std. Deviation	Std. Error Mean	the Difference Lower	the Difference Upper			
Pair 1	VAR00002 - VAR00003	30,41150	16,35289	6,67604	13,25020	47,57280	4,555	5	,006

statistically significant

Test D. ACH measured using CO2 Concentration Increase Method. Natural Concentrations

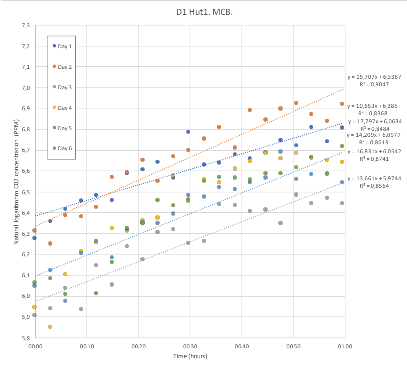

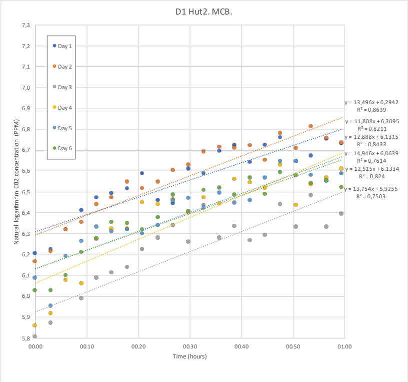

ACH (Air exchange hour)

D1 Hut1. MCB	D1 Hut2. MCB
15,707	13,496
10,653	11,808
17,797	12,888
14,209	14,945
16,831	12,515
13,661	13,754
14,810	13,234

89%

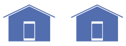

MCB MCB

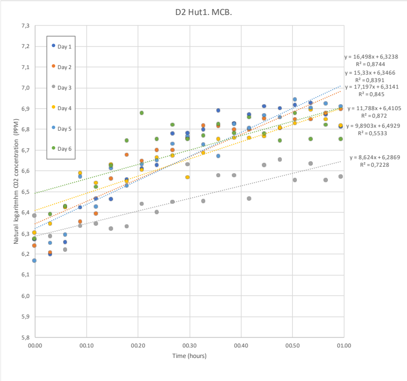

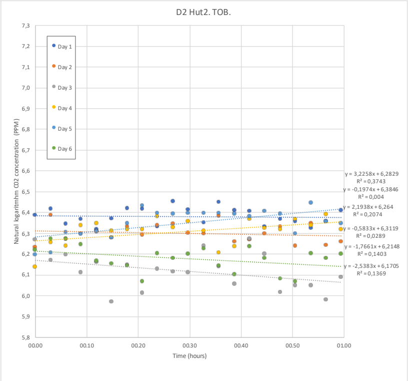

ACH (Air exchange hour)

D2 Hut1. MCB	D2 Hut2. TOB
16,498	3,226
15,330	-0,197
17,197	2,194
11,788	-0,583
9,890	-1,766
8,624	-2,538
13,221	0,056

0%

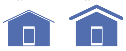

MCB TOB

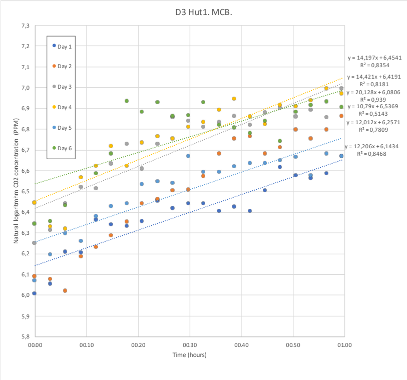

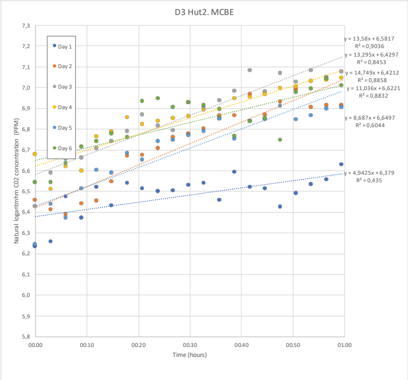

ACH (Air exchange hour)

D3 Hut1. MCB	D3 Hut2. MCBE
14,197	13,580
14,421	13,295
21,128	14,749
10,790	11,036
12,012	8,687
12,202	4,943
14,125	11,048

78%

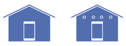

MCB MCBE

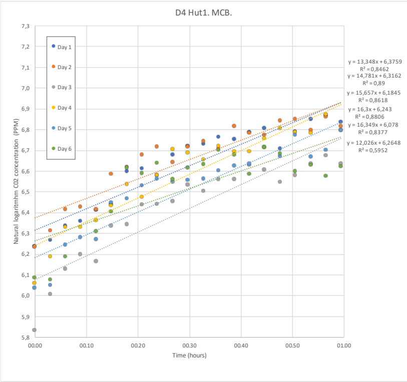

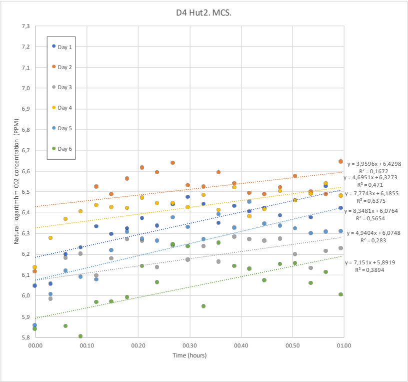

ACH (Air exchange hour)

D4 Hut1. MCB	D4 Hut2. MCS
13,348	3,960
14,781	4,695
15,657	7,774
16,300	8,348
16,349	4,940
12,020	7,151
14,743	6,145

42%

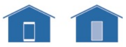

MCB MCS

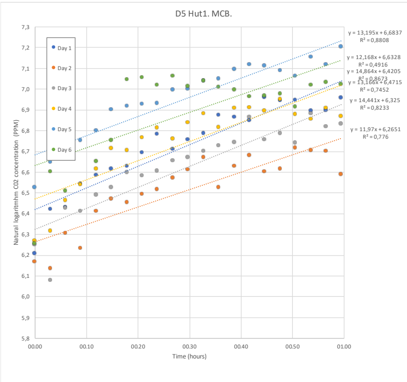

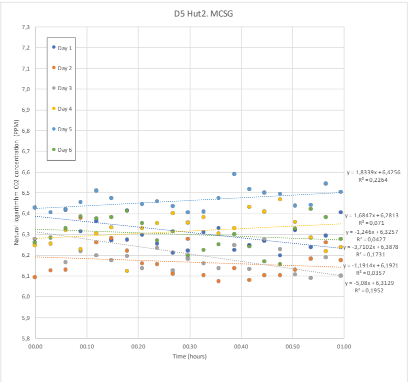

ACH (Air exchange hour)

D5 Hut1. MCB	D5 Hut2. MCSG
13,195	1,834
12,168	1,685
14,864	-1,246
13,166	-3,710
14,441	-1,191
11,970	-5,080
13,301	-1,285

-10%

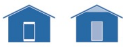

MCB MCSG
