# Supplementary material for: Measuring ventilation in different typologies of rural Gambian houses: a pilot experimental study
Source: Malar J. 2020 Jul 31;19:273. doi: 10.1186/s12936-020-03327-0 (PMC7393878; doi:10.1186/s12936-020-03327-0)
Supplement: Supplementary file 2 — Additional file 2: Table S1. Summary data of air exchange with different typologies of houses. [file 12936_2020_3327_MOESM2_ESM.docx]

Table S1. Summary data of air exchange with different typologies of houses
